# Individual islet respirometry reveals functional diversity within the islet population of mice and human donors

**DOI:** 10.1016/j.molmet.2018.07.003

**Published:** 2018-07-25

**Authors:** Evan P. Taddeo, Linsey Stiles, Samuel Sereda, Eleni Ritou, Dane M. Wolf, Muhamad Abdullah, Zachary Swanson, Josh Wilhelm, Melena Bellin, Patrick McDonald, Kacey Caradonna, Andrew Neilson, Marc Liesa, Orian S. Shirihai

**Affiliations:** 1 Department of Medicine, Division of Endocrinology, Diabetes and Hypertension, and Department of Molecular and Medical Pharmacology, David Geffen School of Medicine at UCLA, Center for Health Sciences, 650 Charles E. Young St., Los Angeles, CA 90095, USA; 2 Department of Medicine, Endocrinology, Diabetes, Nutrition and Weight Management Section, Boston University School of Medicine, 650 Albany St., Room 840, Boston, MA 02118, USA; 3 Department of Surgery and Schulze Diabetes Institute, University of Minnesota School of Medicine, Minneapolis, MN 55455, USA; 4 Department of Pediatrics, Division of Pediatric Endocrinology, University of Minnesota School of Medicine, Minneapolis, MN 55455, USA; 5 Center for Health Research Innovation, University of Alberta, Edmonton, AB T6G 2E1, Canada; 6 Agilent Technologies, Lexington, MA 02421, USA

**Keywords:** Islets, Mitochondria, Respirometry, Glucose, OCR, oxygen consumption rate, Ant A, Antimycin A, Oligo, Oligomycin A, FCCP, Carbonyl cyanide-*4*-(trifluoromethoxy)phenylhydrazone

## Abstract

**Objective:**

Islets from the same pancreas show remarkable variability in glucose sensitivity. While mitochondrial respiration is essential for glucose-stimulated insulin secretion, little is known regarding heterogeneity in mitochondrial function at the individual islet level. This is due in part to a lack of high-throughput and non-invasive methods for detecting single islet function.

**Methods:**

We have developed a novel non-invasive, high-throughput methodology capable of assessing mitochondrial respiration in large-sized individual islets using the XF96 analyzer (Agilent Technologies).

**Results:**

By increasing measurement sensitivity, we have reduced the minimal size of mouse and human islets needed to assess mitochondrial respiration to single large islets of >35,000 μm^2^ area (∼210 μm diameter). In addition, we have measured heterogeneous glucose-stimulated mitochondrial respiration among individual human and mouse islets from the same pancreas, allowing population analyses of islet mitochondrial function for the first time.

**Conclusions:**

We have developed a novel methodology capable of analyzing mitochondrial function in large-sized individual islets. By highlighting islet functional heterogeneity, we hope this methodology can significantly advance islet research.

## Highlights

• Islets from the same pancreas show remarkable variability in glucose sensitivity.
• Little is known about heterogeneity in mitochondrial respiration in individual islets.
• We developed a novel high-throughput method to measure individual islet function.
• Individual islets within a population show heterogeneous glucose-stimulated respiration.
• Our method may advance islet research by enabling populational metabolic profiling.

## 1 Introduction

Pancreatic islets rely on mitochondrial respiration to secrete insulin [1], a critical function for maintaining metabolic homeostasis. A rise in extracellular glucose levels increases TCA cycle flux, mitochondrial respiration, and ATP synthesis in islet β-cells, which generates molecular signals stimulating and amplifying insulin secretion [2]. Indeed, even at the level of the whole organism, mitochondrial respiratory function accounts for approximately 90% of total oxygen consumption, 80% of which is coupled to ATP synthesis [3]. Therefore, measuring oxygen consumption represents the gold standard assessment of metabolic behavior, bioenergetic demand, and mitochondrial function for islet tissue. Furthermore, by using different compounds targeting mitochondria, one can determine respiration linked to mitochondrial ATP synthesis and the maximal capacity of cells oxidizing fuels through the electron transport chain [4].

Islets and β-cells within an islet are known to show remarkable variability in glucose sensitivity [5], [6], [7], [8], [9], [10], size [11], [12], architecture [8], and cell composition [13] among other factors. A small group of “first responder” islets rapidly respond to glucose *in vivo* by releasing almost their entire load of insulin, while some islets remain dormant and remain unresponsive to glucose [7], [14]. Islet heterogeneity also seems to play a critical role in pathogenesis of metabolic disease, as distinct groups of islets are more susceptible to dysfunction in diabetes [13]. In addition, one could predict that islet preparations with a low number of “responder islets” and a high number of “dormant islets” would be less likely to efficiently sustain insulin production after their transplantation into subjects with diabetes. This could be the case since a high proportion of islets do not survive transplantation, and a low number of responder islets would decrease the probability of providing sufficient insulin in response to glucose. Despite the essential role of mitochondrial respiration in insulin secretion [1], [15], very little is known about heterogeneity in mitochondrial function among islets from a given population. Currently, it has not been possible to address this issue due to a lack of high-throughput methods capable of quantifying mitochondrial function of individual islets within a population.

The current gold standard for islet respirometry is the XF24 islet capture plate [16]. Despite being the most sensitive oxygen consumption methodology, the islet capture plate requires 50–80 islets per well, which is almost the entire islet population from one mouse when performed in triplicate. Furthermore, only 20 different conditions can be tested simultaneously. This experimental platform is not suitable for assessing large numbers of biological samples and lacks the sensitivity to detect respiration of individual islets from heterogeneous populations. Other more invasive methodologies can measure oxygen consumption in single islets [17] but require complex microfluidics chips for encapsulating islets after staining with a fluorescent dye. Single islet ATP measurements are possible [18] but require viral transduction of biosensors. These more invasive manipulations have not been developed for high-throughput measurements, potentially limit the detection of endogenous functional heterogeneity, and could preclude islet use for transplantation. Therefore, a rapid, non-invasive, real-time and high-throughput method is needed to quantify islet functional heterogeneity.

In the present study, we established a non-invasive methodology that enables high-throughput measurement of oxygen consumption in large-sized individual islets from mice and humans. We have developed in collaboration with Agilent Technologies a specialized microplate for the XF instrument called the “spheroid plate”. This plate contains a perfusion insert in each well that allows stable media flow across islets during mixing, without perturbing islet function. The spheroid plate reduces the biological sample size required for mitochondrial respiratory analysis and maintains the functionality for bioenergetics measurements currently used with the XF24 islet capture plate. Our new approach allows a populational analysis of metabolic function that revealed heterogeneous glucose sensitivity in large individual islets from both humans and mice. We hope our methodology can accelerate islet research and help elucidate mechanisms underlying the metabolic heterogeneity in pancreatic islets.

## 2 Materials and methods

### 2.1 Materials

Collagenase P, d-glucose, l-leucine, l-glutamine, carbonyl cyanide-4-(trifluoromethoxy)phenylhydrazone (FCCP), and Antimycin A were purchased from Sigma Aldrich (St. Louis, MO). Fatty acid-free bovine serum albumin (BSA) was purchased from EMD Millipore (Billerica, MA). Fetal bovine serum (FBS) was obtained from Life Technologies (Carlsbad, CA). Oligomycin A was obtained from Calbiochem (San Diego, CA). Accutase was purchased from Thermo Fisher Scientific (Roskilde, Denmark). Seahorse XF96 spheroid microplates, Seahorse XF96 FluxPaks, Seahorse XF Calibrant Solution and Seahorse XF Base Medium Minimal DMEM were acquired from Agilent Technologies (Santa Clara, CA).

### 2.2 Isolation and culture of mouse islets

Islets were isolated from 11 to 16 week old male C57BL/6J mice (Jackson Labs, Bar Harbor, ME) via collagenase P injection into the bile duct, as previously described [16], [19]. Islets were cultured overnight at 37 °C 5% CO_2_ in islet media (11 mM glucose RPMI 1640 + 10% FBS + 100 U/mL penicillin, and 100 μg/mL streptomycin) prior to experimentation.

### 2.3 Human islets

Human islets were obtained from the University of Alberta Diabetes Institute Islet Core (Edmonton, Alberta, Canada) in collaboration with Dr. Patrick MacDonald. Islets were isolated from six non-diabetic deceased donors ages 18–71 with 60–95% purity, cultured 7–40 h post-isolation and shipped overnight at 4 °C in CMRL media (Gibco/ThermoFisher, Waltham, MA). Upon arrival, islets were further purified from exocrine pancreas and cellular debris by visual inspection and picking. Islets were cultured overnight in fresh CMRL media at 37 °C and 5% CO_2_ before measurement of mitochondrial respiration.

Human islets were also obtained from the University of Minnesota Schulze Diabetes Institute (Minneapolis, MN, USA) in collaboration with Josh Wilhelm. Islets were isolated from living donors with pancreatitis undergoing total pancreatectomy with islet auto-transplantation (Patient 1: 57 years old, non-diabetic female, BMI = 25, HbA1c = 4.8%; Patient 2: 57 years old, non-diabetic male with hyperlipidemia, BMI = 23, HbA1C = 5.8%). Islets were shipped overnight (4 °C, with Cryopaks) in Transplant Media (CMRL supplemented with 2.5% human serum albumin, 25 mM HEPES and 20 μg/mL ciprofloxacin). Upon arrival, islets were further purified from exocrine pancreas and cellular debris by visual inspection and picking. Islets were then cultured for 1–2 h at 37 °C 5% CO_2_ in CMRL media supplemented with 10 mM niacinamide, 1% (v/v) insulin-transferrin-selenium, 16.7 μM ZnSO_4_, 5 mM sodium pyruvate, 1% (v/v) Glutamax, 25 mM HEPES, 10% (v/v) FBS, and 1% (v/v) pen/strep. Respirometry was conducted on the same day that the islets arrived at UCLA. Approximately 1–2% of the final isolated islet product from pancreatitis patients was used for experiments.

### 2.4 XF96 spheroid plate islet respirometry

Mouse or human islets (1–32 islets/well) were seeded into wells of a poly-d-lysine-coated (100 μg/mL) XF96 spheroid plate containing 100–175μL/well of warm assay medium (Seahorse XF base medium minimal DMEM, supplemented with 3 mM glucose and 0.1% FBS). Islet seeding was done by aspirating islets in a minimal volume of media (∼4–15 μL) and inserting the pipette tip into each well of the spheroid plate, using a Leica S6E microscope to orient the pipette tip directly over the central depression in the well. Islets fall out of the pipette tip by gravity and into the central detent of each well. Once seeded, islets in the plate were centrifuged at 450 rpm for 7 min with no centrifuge brake, then incubated for 1–2 h at 37 °C in a non-CO_2_ incubator. Mitochondrial respiration was measured using the Seahorse XF96 extracellular flux analyzer equipped with a spheroid plate-compatible thermal tray (Agilent Technologies, Santa Clara, CA). Basal respiration was first measured in 3 mM glucose media. Islets were then sequentially exposed to glucose (final concentration in well of 20 mM), Oligomycin A (3.5–4.5 μM final concentration), FCCP (1 μM final concentration) and Antimycin A (Ant A, 2.5 μM final concentration).

To validate individual islet respirometry with the XF96 spheroid plate, individual mouse islets were exposed to either 20 mM glucose or glucose + amino acids (AA, 10 mM each of leucine and glutamine), followed by FCCP (final concentration of 1 μM) and Antimycin A (final concentration of 3 μM). For assays testing reproducibility, single islets were seeded in a spheroid plate as described above, and basal respiration in 3 mM glucose media was measured until steady state respiration was achieved. Islets were then transferred to different wells in a new spheroid plate and steady state basal respiration was measured again. For assessment of islet functional heterogeneity, basal respiration of individual islets was first measured in 3 mM glucose media. Islets were then acutely exposed to 20 mM glucose for approximately 60–90 min, followed by Antimycin A.

### 2.5 XF24 islet capture plate respirometry

Respirometry using the Seahorse XF24 was performed as previously described [16], with pooled islets (50–80 islets/well). For direct comparisons between the XF96 spheroid and XF24 islet capture plates, mouse islets from the same islet isolation or human islets from the same donor were used for experiments.

### 2.6 Calculations for islet bioenergetics

For calculation of mouse and human islet bioenergetics parameters, average non-electron transport chain OCR values after injection of Antimycin A were subtracted from all OCR measurements. All parameters were normalized to basal respiration and reported as % of basal OCR. Glucose-stimulated respiration was calculated by dividing the first OCR measurement after injection of 20 mM glucose by the last basal OCR measurement and multiplying by 100. Proton leak was calculated by dividing the minimum OCR value after injection of Oligomycin A by the last basal OCR value and multiplying by 100. Maximal respiration was determined by dividing the first measurement after FCCP injection by the last basal OCR value and multiplying by 100.

### 2.7 Quantification of islet area

After oxygen consumption measurement, individual islets in the XF96 spheroid plate were imaged in brightfield (10× objective) with the Operetta high-throughput imaging system (Perkin Elmer, Waltham, MA). Islet images were analyzed in ImageJ by manually drawing a line around the perimeter of each islet with the “Polygon selection” drawing feature and selecting the “Measure” function under the “Analyze” tab.

### 2.8 Islet dispersion and reaggregation

Human or mouse islets were transferred to a 15 mL conical tube and centrifuged at 1000 rpm for 2 min. The supernatant was aspirated, and islets were dissociated by adding accutase (1 mL accutase/1000 islets) and incubating for 10 min at 37 °C. After 10 min, islets were pipetted up and down gently to facilitate dissociation. Islet cells were transferred to a new tube, and the accutase was quenched with 15 mL of islet media. The islet cell suspension was strained with a 40 μm cell strainer (Fisher Scientific, Waltham, MA) and centrifuged at 1000 rpm for 5 min. The supernatant was aspirated, and the cell pellet was resuspended in 5 mL of islet media. Cells were counted and seeded at 6,000, 8,000 or 10,000 cells per well of a V-bottom 96 well plate (Thermo Fisher Scientific, Roskilde, Denmark). Plates were centrifuged at 1000 rpm for 5 min and incubated at 37 °C 5% CO_2_ for 48–72 h before experiments.

### 2.9 Statistics

Data were expressed as an individual islet OCR trace or as means ± standard error of the mean (SEM) or standard deviation of islets derived from 1 to 5 human donors or 1–4 independent mouse islet isolations. p-values were calculated by two-way ANOVA with Sidak's multiple comparisons test or linear regression. Statistical significance was set at p < 0.05.

### 2.10 Data availability

The datasets generated during and/or analyzed during the current study are available from the corresponding author on reasonable request.

### 2.11 Study approval

All animal care was in accordance with NIH guidelines and the University of California Los Angeles institutional animal care and use committee (Chancellor's Animal Research Committee, UCLA protocol #16-018). Mice used for islet isolations were housed at University of California Los Angeles Animal Resources Facility. Mice were housed at a maximum of 5 per cage and maintained on a 12 hr/12 hr light/dark cycle at 68–72 °F. Mice were provided with water and food *ad libitum* until the day of islet isolation. Human samples were collected from participants in a prospective single center IRB-approved protocol on total pancreatectomy and islet auto-transplant at the University of Minnesota. Informed consent was obtained from study participants for general study participation and for pancreatic tissue analyses.

## 3 Results

### 3.1 Design of the XF96 spheroid plate for high-throughput islet respirometry

Oxygen consumption can be measured in islets using the XF24 islet capture plate; however, this method requires a significant number of islets per experimental replicate and is not high-throughput [16]. As a consequence, current respiration measurements average large quantities of islets that may contain individual islets that display unique characteristics. The Seahorse XF96 extracellular analyzer has the potential to be a suitable platform to develop a high-throughput methodology for individual islet respiration. In contrast to the current XF24 method, the XF96 has sufficient sensitivity to detect respiration in a few thousand cells and has 96 wells instead of 24 wells. However, there are a few challenges that need to be addressed. The ideal 96 well plate format should: 1) confine the islet within the central region of the well and maintain its proximity to the oxygen sensor, 2) generate a transient small volume measurement chamber to increase signal to noise ratio, 3) protect the islet from oxygen probe-induced damage, and 4) allow free movement of islet cell culture media across the whole islet during media mixing.

**Figure 1:**
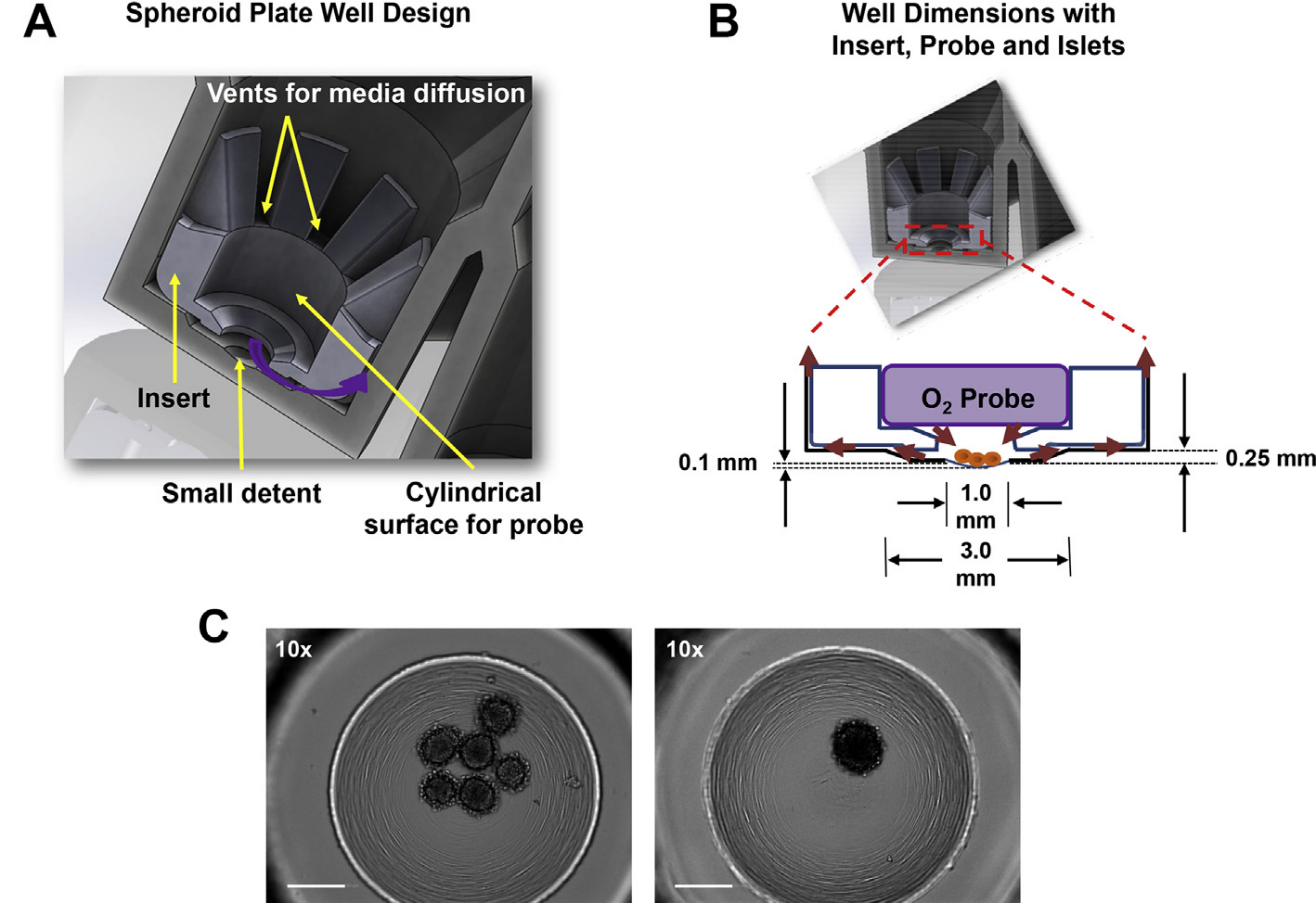
**Design of the XF96 spheroid plate for high-throughput islet respirometry**. (A) Cross-sectional diagram depicting the design of a single well of the new XF96 spheroid plate. Yellow arrows highlight the small detent for islets, configuration of the well insert, space for the oxygen probe and vents for media diffusion. Purple arrow indicates media flux between the small measurement chamber containing the islet and the rest of the well. (B) Dimensions of the XF96 spheroid well, showing the small volume measurement chamber containing islets (small brown spheres) created when the oxygen probe sits in the well insert. (C) Operetta brightfield images of 6 mouse islets or an individual mouse islet in a well of the XF96 spheroid plate after a Seahorse respirometry experiment. Images captured at 10× magnification. Scale bars = 200 μm.

To achieve these goals, two major modifications to the current XF96 plate were introduced, and the new resulting plate was named the “spheroid plate” (Figure 1A). On the surface of the well, a small semispherical detent (1 mm in diameter and 0.1 mm depth) was added to confine islets within the central area of the well, aided by poly-d-lysine coating (Figure 1A–B). The small detent is located inside a larger circular and flat detent (3 mm in diameter and 0.25 mm depth) (Figure 1B), which holds the new insert (Figure 1A–B). The shape of the insert is specifically designed to generate a small volume measurement chamber (approximately 2 μL) below the oxygen probe and facilitates efficient media interchange with the rest of the well through eight narrow vents (Figure 1A). The distance between the vents and the well wall is 0.04 mm (Figure 1B), through which media moves laterally and vertically during oxygen probe-mediated mixing (Figure 1B, red arrows). The vents are narrow to limit significant oxygen exchange rates between the measurement chamber and the rest of the well when the oxygen probe is in measurement position (Figure 1B). Thus, the novel spatial configuration formed by the insert and the large detent is designed to decrease the volume of the chamber during oxygen tension measurements and to maintain the islet in the center of the well during mixing.

Islets are seeded in each well of a spheroid plate by aspirating the desired number of islets in a low volume (∼4–15 μL) of media with a pipette and carefully placing the islets in the detent at the bottom of the well filled with 100–150 μL of pre-warmed Seahorse assay media. A microscope is used to situate the pipette tip directly above the central detent in the middle of the well. Once in position, the islets will fall out of the pipette tip and rest in the central detent within the measurement zone. Using mouse islets, we validated the capacity of the XF96 spheroid plate to accurately measure islet oxygen consumption (Supplementary Figure 1). Figure 1C shows six mouse islets and an individual mouse islet situated in the central detent of a well in the new XF96 spheroid plate after measuring respiration. In most cases, the islets remain intact and are not damaged by the oxygen probe during the mixing steps of a Seahorse assay (Figure 1C). Unlike the XF24 islet capture plate, the well structure of the spheroid plate obviates the mesh net required to trap islets in the XF24 well measurement chamber. This facilitates the islet loading procedure and removes a potential source of measurement variability.

### 3.2 The XF96 spheroid plate is capable of measuring respiration in large individual human and mouse islets

**Figure 2:**
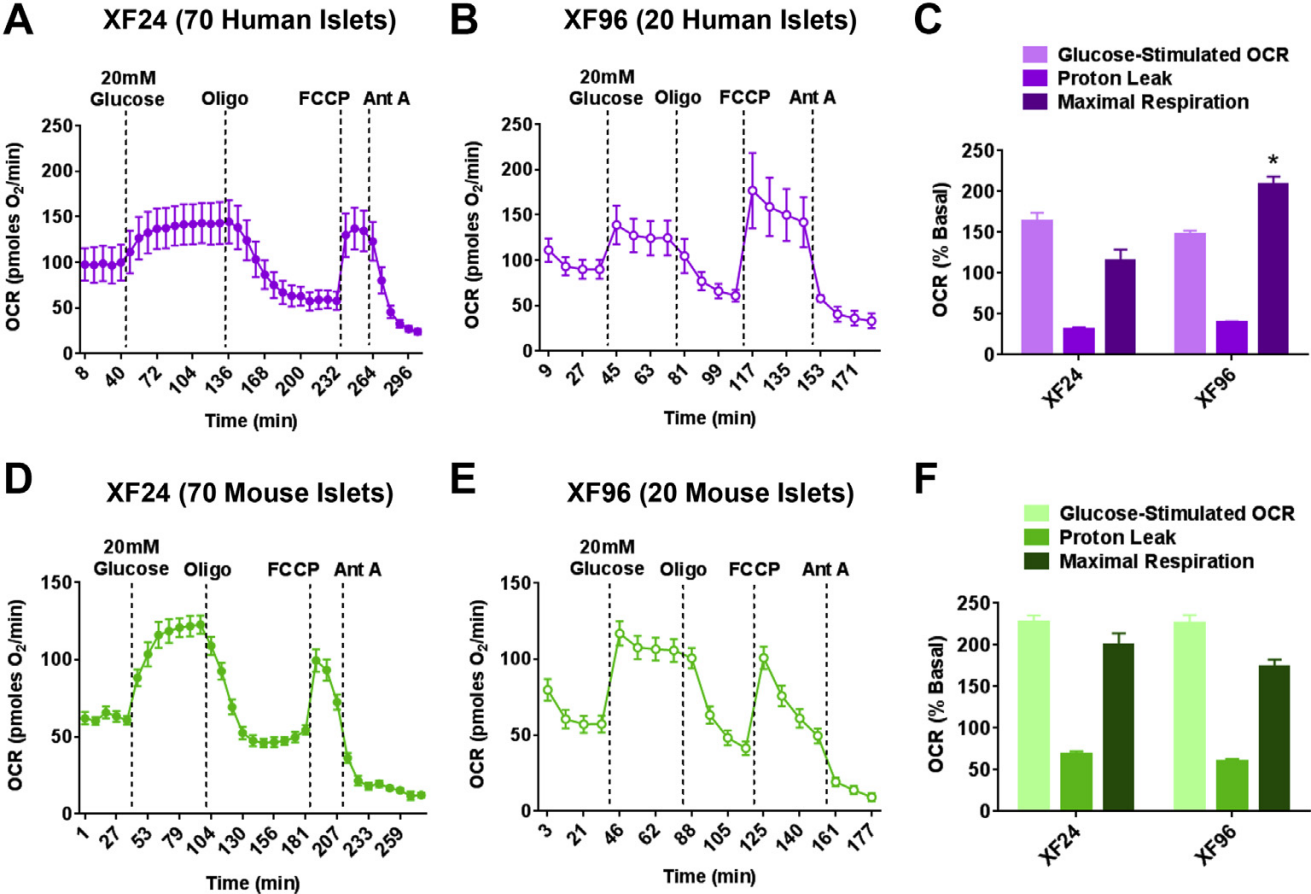
**The XF96 spheroid plate is capable of measuring respiration in small samples of human and mouse islets**. (A) Representative oxygen consumption rate (OCR) traces of human islets measured with the XF24 islet capture plate. Islets (70 per well) were acutely exposed to 20 mM glucose (final concentration), Oligomycin A (Oligo), FCCP, and Antimycin A (Ant A). n = 6 wells from one donor islet preparation. (B) Representative OCR traces of human islets measured with the X96 spheroid plate. Islets (20 per well) were acutely exposed to the same compound injections as in (A). n = 3 wells from the same human donor islet preparation as in (A). (C) Glucose-stimulated OCR, proton leak and maximal respiration from human islets measured in the XF24 islet capture plate or the XF96 spheroid plate. n = 5 independent experiments from 5 human donors. *p < 0.05 compared to XF24 maximal respiration by two-way ANOVA. (D) Representative XF24 OCR traces of 70 mouse islets per well. n = 4 wells from one islet isolation. (E) Representative OCR traces of 20 mouse islets per well measured with the X96 spheroid plate. Islets were from the same islet preparation as measured in (D). n = 8 wells from one islet isolation. (F) Glucose-stimulated OCR, proton leak and maximal respiration from mouse islets measured in the XF24 islet capture plate or the XF96 spheroid plate. n = 3 independent experiments for XF96 and 1 experiment for XF24. Human islets in (A–C) were obtained from deceased donors in collaboration with University of Alberta Diabetes Institute Islet Core. Data are means ± standard error of the mean (SEM).

The XF24 islet capture plate is capable of measuring respiration in human islets [16], but the required large sample size and low throughput of this technique limit its potential as a tool for detecting islet heterogeneity in a population. To investigate whether the XF96 spheroid plate may be a suitable high-throughput platform for individual human islet respirometry, we directly compared mitochondrial respiration measurements with either the XF24 or the XF96 methods using the same human islet preparations. Using 70 human islets per well, the XF24 yielded stable OCR measurements and showed expected responses to stimulatory glucose, Oligomycin A, FCCP, and Antimycin A (Figure 2A). Based on the difference in well size between the XF24 and XF96 instruments, we initially tested 20 humans islets per well in the spheroid plate. The XF96 spheroid plate produced stable OCR traces similar to those of the XF24 (Figure 2B), indicating that the XF96 platform can perform human islet respirometry with less than half of the islets required with current XF24 technology. When comparing results from five human donors, glucose-stimulated respiration and proton leak were similar between the XF24 and XF96 methods (Figure 2C), but human islets in the spheroid plate showed a significantly higher maximal respiration in the presence of FCCP (Figure 2C) compared to same islets measured in the XF24.

We then determined whether we could reduce the sample size required for bioenergetics assessment of mouse islets using the spheroid plate. To accomplish this, we performed respiratory analyses with 20 mouse islets in each XF96 well and directly compared them to the respiratory traces obtained using 70 mouse islets per well of the XF24 islet capture plate (Figure 2D). The XF96 spheroid plate produced very similar OCR traces compared to those generated with the XF24 (Figure 2E), revealing nearly identical glucose-stimulated respiration, proton leak and maximal respiration (Figure 2F). We also performed a titration in a wider range of islet number per well and found that the spheroid plate could detect respiration in a single islet (Supplementary Figure 2).

**Figure 3:**
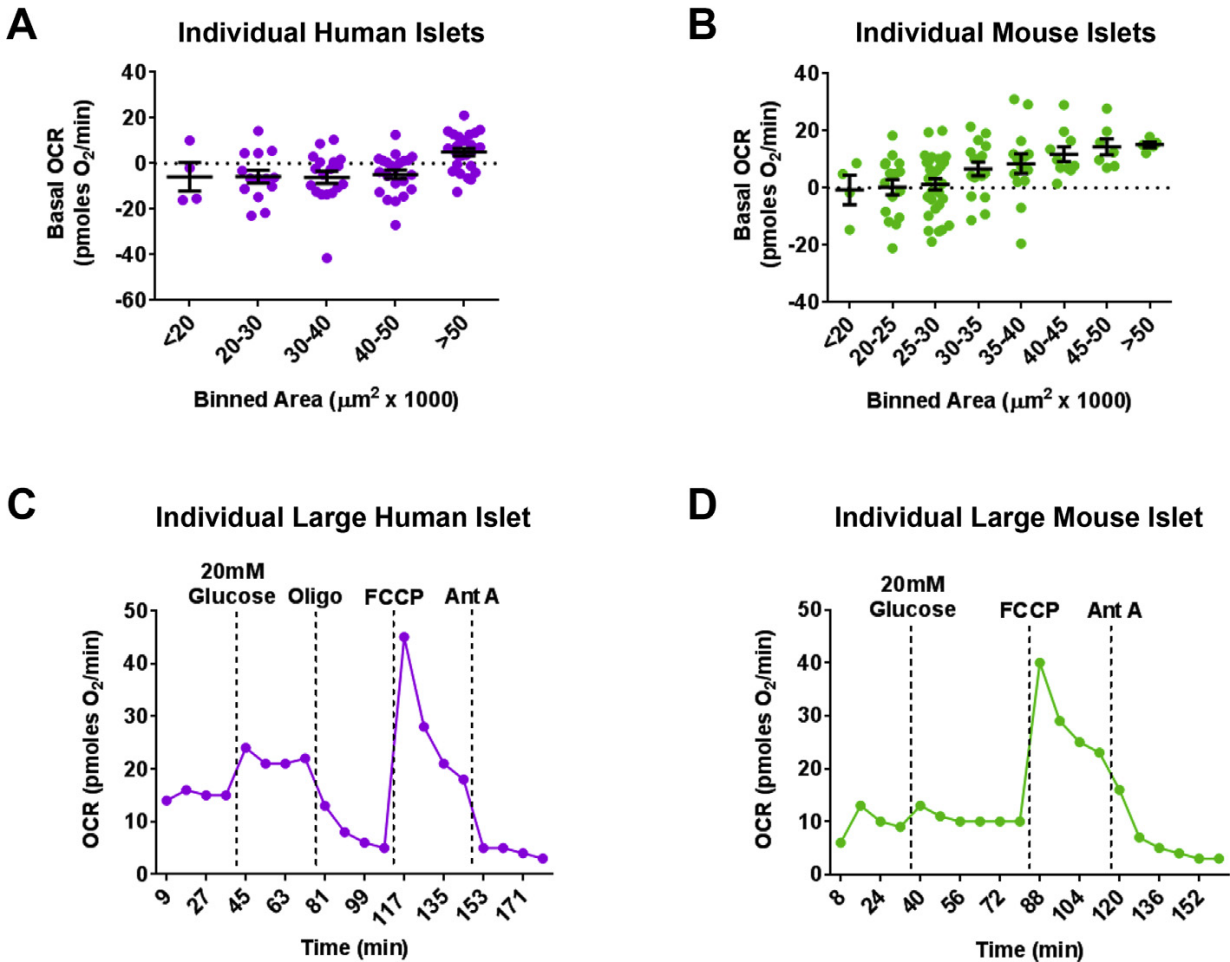
**Individual human and mouse islet respirometry with the XF96 spheroid plate**. (A) Spheroid plate measurements of basal OCR of different-sized individual human islets. Islets were binned according to area, from <20,000 μm^2^ (small) to >50,000 μm^2^ (large). Non-electron transport chain OCR is subtracted from the positive respiratory values, but not for the negative values. Black bars indicate mean and SEM. n = 84 islets from 2 donors. (B) Basal OCR of different-sized individual mouse islets. Islets were binned according to area, and OCR was adjusted as noted for (A). Black bars indicate mean and SEM. n = 101 islets from 4 independent experiments. (C) Representative OCR trace of a large individual human islet exposed to 20 mM glucose, Oligomycin, FCCP, and Antimycin A measured with the spheroid plate. (D) Representative OCR trace of a large individual mouse islet exposed to 20 mM glucose, FCCP, and Antimycin A measured with the spheroid plate. Human islets were obtained from living donors with pancreatitis undergoing total pancreatectomy with islet auto-transplantation at the University of Minnesota (A), and from deceased donors from University of Alberta Diabetes Institute Islet Core (C). Data in (A–B) are means ± SEM.

The spheroid plate drastically reduced the sample size required for islet bioenergetics (Supplementary Figure 2), but characterization of islet heterogeneity requires the sensitivity to measure a population of individual islets. In addition, the absence of a clear linear response in OCR vs. islet number at the range between 1 and 6 islets/well suggested that respiration measurements were not possible in every individual islet. To this end, we investigated whether the spheroid plate had the sensitivity to consistently profile bioenergetics of individual islets from several different human donors and mouse pancreata, as well as the characteristics of the individual islets in which respiration was successfully detected. Because islets vary in size even in the same pancreas [11], [12], we measured basal OCR in individual human (Figure 3A) and mouse (Figure 3B) islets of different sizes, with areas <20,000 μm^2^ (small islets, less than ∼170 μm diameter) to >50,000 μm^2^ (large islets, greater than ∼290 μm diameter). The spheroid plate detected basal respiration in single human islets of all sizes, but many of the islets with an area under 50,000 μm^2^ showed basal OCR values below background signal (Figure 3A). However, the majority of human islets with an area greater than 50,000 μm^2^ yielded positive basal respiratory values (Figure 3A). Similar to human islets, the spheroid plate detected OCR from single mouse islets with an area over 35,000 μm^2^ (diameter greater than ∼ 210 μm) (Figure 3B). Importantly, the detected differences in OCR among different-sized islets were not a consequence of islet displacement from the central measurement area of the well (Supplementary Figure 3).

To further explore the capability of the spheroid plate to monitor changes in mitochondrial metabolism at the individual islet level, we subjected large-sized single human (Figure 3C) and mouse (Figure 3D) islets to 20 mM glucose, Oligomycin, FCCP, and Antimycin A. Remarkably, individual large-sized human islets yielded OCR traces similar to those produced by the spheroid plate with 20 islets per well in Figure 2. Glucose and FCCP stimulated respiration while Oligomycin and Antimycin A inhibited respiration (Figure 3C), enabling calculation of glucose sensitivity, proton leak and maximal respiration in individual human islets (Supplementary Figure 4). Importantly, the spheroid plate was able to detect nutrient- and mitochondrial drug-induced changes in respiration of large individual mouse islets (Figure 3D), allowing for population bioenergetics in islets from mice as well (Supplementary Figure 4). These results indicate that the XF96 spheroid plate has the sensitivity to consistently assess respiratory function in single mouse islets larger than 35,000 μm^2^ in area (∼210 μm diameter) and individual human islets larger than 50,000 μm^2^ area (∼290 μm diameter).

### 3.3 The XF96 spheroid plate reveals heterogeneity in glucose-stimulated respiration in individual human and mouse islets

**Figure 4:**
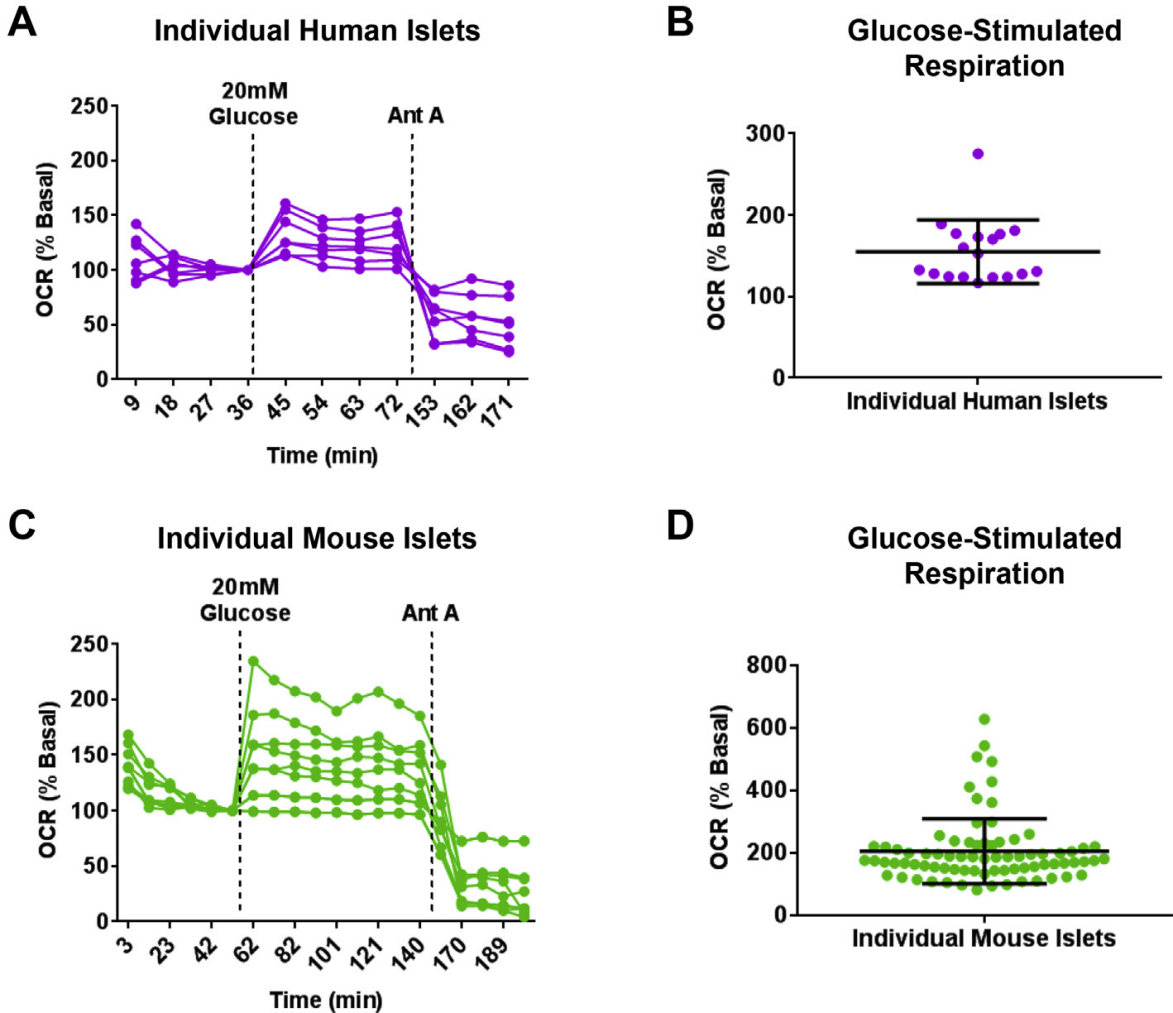
**The XF96 spheroid plate reveals heterogeneity in glucose-stimulated respiration in individual human and mouse islets**. (A) Representative traces of glucose-stimulated respiration in large individual human islets measured with the spheroid plate. Data are normalized to basal respiration (% Basal). Each trace represents one islet. n = 7 islets from 3 donors. (B) Quantification of glucose-stimulated respiration in single human islets measured in the spheroid plate. Each data point is an individual islet. Black bars indicate mean ± standard deviation (SD). n = 18 islets from 4 donor islet preparations. (C) Representative traces of glucose-stimulated respiration in large individual mouse islets measured with the spheroid plate. Data are normalized to basal respiration (% Basal). Each trace represents one islet. n = 10 islets. (D) Quantification of glucose-stimulated respiration in single mouse islets measured in the spheroid plate. Each data point is an individual islet. Black bars indicate mean ± SD. n = 77 islets from 4 independent experiments. Human islets in (A–B) were obtained from deceased donors from University of Alberta Diabetes Institute Islet Core.

Islets from the same pancreas show remarkable variability in glucose sensitivity [7], [20]; however, little is known regarding the heterogeneity in glucose-stimulated mitochondrial respiration at the individual islet level. To assess variability in mitochondrial responses to glucose, we measured glucose-stimulated respiration in large individual human and mouse islets using the spheroid plate. Glucose stimulation revealed notable heterogeneity in respiration among both large-sized individual human (Figure 4A) and mouse (Figure 4C) islets. Large-sized individual human islets showed variable glucose sensitivity, with an average increase in respiration of roughly 150% of basal levels (Figure 4B). However, while some large-sized islets did not respond to glucose, one islet increased respiration over 250% of basal rates (Figure 4B). The majority of large-sized individual mouse islets increased oxygen consumption approximately 200% or less compared to basal in the presence of stimulatory glucose (Figure 4D). A few islets showed either no response to glucose or a very high glucose response reaching up to 600% of basal respiration (Figure 4D), indicating heterogeneity in glucose-activated mitochondrial activity among large-sized individual human and mouse islets. This spectrum of glucose sensitivity was also observed in glucose-stimulated insulin release measured from individual intact mouse islets in the spheroid plate (Supplementary Figure 5).

**Figure 5:**
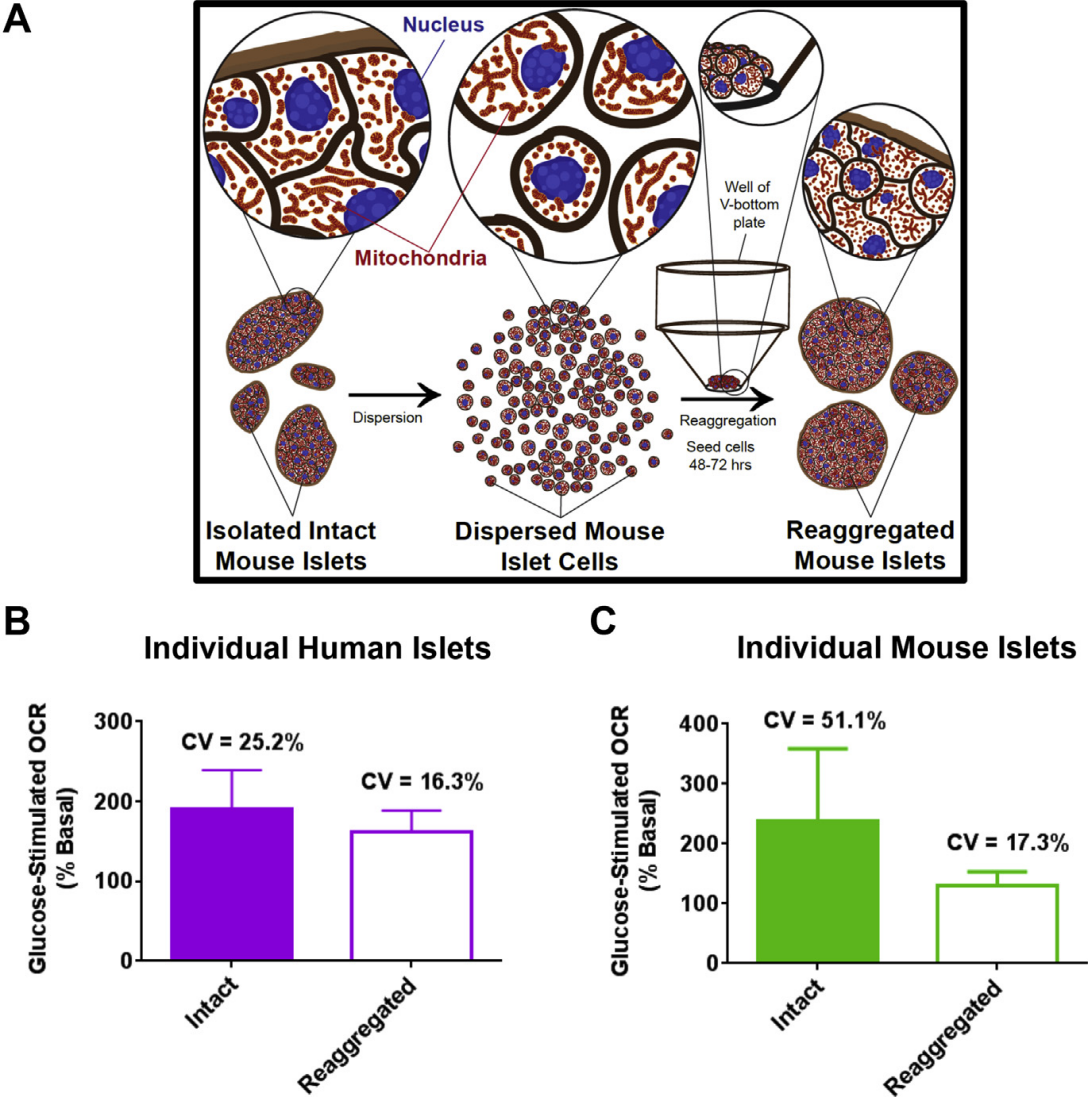
**Dispersion and reaggregation of islet cells reduces heterogeneity in glucose-stimulated respiration among individual islets**. (A) Dispersion and reaggregation of islets. Isolated mouse islets were cultured overnight, pooled and dispersed with accutase into single cells. Single islet cells were seeded at 6,000–10,000 cells/well and cultured for 48–72 h in V-bottom 96 well plates to facilitate reaggregation into islet structures (reaggregated islets). (B) Glucose-stimulated respiration of individual intact or reaggregated human islets, normalized to basal OCR (% Basal). Intact or reaggregated human islets were derived from the same donors. n = 5 intact islets and 7 reaggregated islets from 2 donors. (C) Glucose-stimulated respiration of individual intact or reaggregated mouse islets, normalized to basal OCR (% Basal). Intact and reaggregated islets were derived from the same islet preparation. n = 8 intact and 7 reaggregated islets from one islet isolation. Coefficient of variation (CV) was calculated as an estimate of variability. Human islets in (B) were obtained from deceased donors from University of Alberta Diabetes Institute Islet Core. Data in (B–C) are means ± SD.

Islets vary in size [11], [12], cell composition [13], and architecture [8], which are important factors that determine islet function. In fact, islets exhibit an appreciable degree of heterogeneity in mitochondrial metabolism [5], an effect that may be due to disparity in islet size and/or cell composition. To ensure that the differences in glucose-stimulated respiration were not due to an assay artifact, we reconstituted islets *in vitro* to form pseudo, or “reaggregated islets.” This manipulation simultaneously normalizes the cell composition and controls the size among the formed islets, facilitating further interrogation of the sensitivity of the XF96 platform. Islets from the same pancreas were pooled, dispersed into single cells and allowed to reaggregate over the course of 48–72 h (Figure 5A). The spheroid plate detected respiration in a single reaggregated islet of 6000 cells (Supplementary Figure 6). The statistically significant linear correlation (R^2^ = 0.999) between the number of plated islet cells and OCR (Supplementary Figure 6) demonstrates that the XF96 spheroid plate can discriminate changes in basal respiration corresponding to an increase of 2000 cells in islet size. Notably, dispersion and reaggregation of both human and mouse islets reduced the variability in glucose-stimulated respiration compared to intact islets (Figure 5B–C and Supplementary Figure 6), suggesting that the heterogeneity in glucose-stimulated respiration among individual islets was not an artifact of the spheroid plate.

## 4 Discussion

Mitochondrial respiration is essential for pancreatic islet insulin release [1], a process that maintains blood glucose levels in response to nutrient consumption. The decline or absence of insulin release has dire metabolic consequences, including severe hyperglycemia and diabetes. However, these pathophysiological changes in islet secretory function do not occur to all islets uniformly [13]. Surprisingly, little is known about the mechanisms underlying this functional heterogeneity at the level of an individual islet and whether this heterogeneity extends to mitochondrial respiratory capacity. This gap in knowledge may be explained by technical limitations, since it is currently not possible to measure mitochondrial function of single islets from a population in a high-throughput and non-invasive manner. In this study, we have developed a novel high-throughput method sensitive enough to detect mitochondrial function at the level of the large individual islet, facilitating populational characterization of islet heterogeneity in a non-invasive manner for the first time. Structural modifications made to the spheroid plate increase measurement sensitivity allowing for mitochondrial respiratory analysis of biological samples down to a large-sized intact single islet with an area >35,000 μm^2^ (diameter ∼210 μm) and a reaggregated islet of 6000 cells. This technological advance enabling metabolic profiling at the individual islet level has been utilized to show heterogeneous glucose sensitivity of mitochondrial respiratory function in both human and mouse islets. Through populational assessment of islet respiration, we hope our method may be used as a research tool to better understand islet function in health and disease.

The new design of the XF96 spheroid plate significantly improves the capability of measuring islet respiratory function compared to the XF24 islet capture plate. The new spheroid microplate employs a well insert that creates a small enough chamber with adequate media flow to maintain islet function and measure oxygen consumption with the Seahorse XF96 analyzer. This insert prevents the requirement of placing a net on top of the well to retain islets within the measurement chamber, a manual and tedious step required for the XF24 method. The spheroid plate well modifications simultaneously reduce the number of islets required per well for accurate measurements, increase the throughput of islet bioenergetics and decrease the time required to obtain stable respiratory traces when compared to the XF24. Mouse and human islet preparations can now be subjected to more experimental conditions and test compounds to probe mitochondrial function. In fact, high-throughput platforms have already been described for drug screening in β-cell spheroids [21], for single cultured cells in sealed sub-nanoliter chambers [22] and in cancer cell spheroids with the spheroid plate [23], but not in islets. Ideally, high-throughput islet respirometry should also preserve functionality throughout the course of an experiment and be amenable to downstream analyses. The XF96 method improves the kinetics of OCR measurement compared to the XF24 and reduces the number of data points needed to achieve stable glucose-stimulated, Oligomycin-resistant and non-mitochondrial respiration in both human and mouse islets (Figure 2). Average assay times for the XF96 spheroid plate were roughly 180 min, compared to approximately 300 min for the XF24 islet capture plate. Shorter overall assay times increase the chances for functional islet assays after respirometry, reducing their time in culture. Despite this reduction in measurement times, the spheroid plate still shows similar capacity for bioenergetics measurements compared to the current XF24 gold standard method for bulk islet respirometry. Thus, we show that the XF96 spheroid plate allows high-throughput measurement of mitochondrial function in intact islets retaining the cellular architecture so critical for their physiological function [11], [13].

Until recently, most islet analyses were performed on larger batches of islets, masking the notable functional heterogeneity that has been uncovered by many groups [5], [6], [7], [10], [12], [24]. For the first time, our spheroid plate facilitates high-throughput mitochondrial respiration profiling of individual human and mouse islets. In fact, full mitochondrial stress test profiles can be generated with large single islets, yielding information on glucose sensitivity, ATP production and proton leak and maximal mitochondrial respiratory capacity for an islet population. Previous studies have introduced a microfluidic-based technology enabling detection of oxygen levels in encapsulated single islets using an oxygen sensitive dye [17]. However, our novel spheroid plate-based technology is minimally invasive, does not require any dyes, and detects individual islet mitochondrial respiration in a high-throughput manner. Furthermore, mitochondrial stressors can be omitted to solely study glucose-induced respiration and collect the islets after the assay for other purposes (i.e. transplantation).

Our new methodology revealed considerable heterogeneity in mitochondrial function among large individual islets from the same pancreas. Islets from a given pancreas greatly range in size [11] and are known to exhibit unique metabolic characteristics [5], [10], [25], [26], [27]. Furthermore, even diversity in mitochondrial function within β-cells of a single islet is a well-documented phenomenon that can dictate cellular metabolism [1], [27], [28]. However, very little is known about heterogeneity in mitochondrial metabolism at the individual islet level. In this study, we observed notable diversity in mitochondrial responses to acute glucose stimulation among large individual human and mouse islets (Figure 4). Some islets were very sensitive to a rise in glucose, while other islets showed little or no increase in mitochondrial oxygen consumption, supporting the notion of unique groups of islets with variable glucose sensing capabilities. In fact, these differences exist *in vivo*, as a small group of “first responder” islets rapidly respond to glucose by releasing almost their entire load of insulin, while most islets remain dormant [7]. It is possible that the observed variability in glucose-stimulated OCR could be due in part to differences in islet cell composition [13], architecture [8], or central islet cell necrosis [29], since these parameters are critical for determining islet capacity for glucose response. Importantly, normalization of islet cellular composition and size through dispersion and reaggregation reduced the heterogeneity in glucose sensitivity observed among individual islets, suggesting that the unique glucose responses detected were not an artifact of the spheroid plate. However, further work is required to fully understand the factors that contribute to metabolic heterogeneity at the single islet level.

High-throughput measurement of individual islet oxygen consumption highlighting functional heterogeneity may represent the first step for a critical biomedical breakthrough. This type of technology can potentially provide a novel parameter to be tested as a quality control measure of islets used for transplantation. Previous studies demonstrated a positive correlation between islet OCR and maintenance of islet function in patients after islet transplantation [30]. Currently, islet transplantations allow type 1 diabetes patients to produce insulin independently for approximately 1–2 years, with diminishing success 2–5 years after transplantation [31], [32]. A major challenge in islet transplantations is that their success rate is hard to predict [33], [34]. Moreover, insulin secretory capacity measured in donated islets *ex vivo* does not correlate with maintenance of islet function and viability within the recipient [29], [33], [35]. It is possible that islet preparations containing a low number of highly sensitive “responder islets” and a high number of “dormant islets” would be less likely to efficiently sustain insulin production after their transplantation, given that a moderate proportion of islets do not survive transplantation due to issues with engraftment, host immune response, and drugs administered post-transplant [35], among other factors. Yet, the current methods for islet quality control analyze pooled islets and would not be able to identify the islet heterogeneity in donor preparations. Despite the inherent challenges of islet transplantation, one could expect that transplanting a higher number of functional islets might prolong endogenous insulin production in diabetics. Thus, detection of single islet heterogeneity in a high-throughput and non-invasive manner may eventually make the spheroid plate a suitable option for assessing human islets before transplantation, considering the thousands of islets needed for a single successful transplant [33], [36]. To prove its utility for islet transplantation, further studies should be performed to test whether glucose-responsive islets selected by the spheroid plate improve clinical outcomes in rodents and patients after islet transfusions.

In addition, there are other important caveats that need to be considered if the XF96 method should be adopted as a biomedical tool. The XF96 spheroid plate can measure large individual human and mouse islets, but it is not sensitive enough to yield respiratory rates above background levels for a mouse islet with an area <35,000 μm^2^ or a human islet with an area <50,000 μm^2^ (Figure 3). This presents a challenge, considering smaller islets can be functionally distinct from large islets [12] and are superior in islet transplantations [29]. However, this challenge may be overcome by pooling the small islets in groups of ∼20 per well. Also, our methodology involves loading of islets one well at a time, which reduces the speed and efficiency of workflow. This procedural bottleneck may be overcome by automating the islet loading process prior to Seahorse respirometry. Finally, once islets with low or high glucose sensitivity are identified, they must be rapidly sorted for down-stream analyses or transplantation. While high-throughput islet sorting is currently possible [37], this technology would have to be integrated with the XF96 spheroid plate in order to maximize applicability of single islet respirometry for islet transplantation.

Overall, the XF96 spheroid plate can be a valuable tool for islet research. The new spheroid plate has the sensitivity to detect respiration in larger individual pancreatic islets in a 96 well microplate format, reducing biological sample size and increasing measurement throughput compared to the XF24 islet capture plate [16]. In addition, the new well insert produces a confined measurement zone which is advantageous not only for islets but for other spheroids in which adherence to the well bottom could pose a significant problem. Individual islet respirometry now permits separation of a heterogeneous islet population into glucose-responsive islets and non-responsive islets, facilitating the study of mechanisms underlying glucose sensitivity and enabling the identification of healthy islets for transplantation. Aside from glucose, the spheroid plate can also be used to detect heterogeneous islet function in response to other nutrients, small molecule therapeutics and genetic perturbations. Thus, our novel populational islet bioenergetics method may be ideal for uncovering mechanisms dictating heterogeneity in islet function and could be further developed into a quality control metric prior to islet transplantation into diabetic patients.

## Author contributions

ML, OSS, LS, and SS conceived this study. EPT, SS, ER, ML, and LS designed and performed the experiments. KC and AN designed the plate, helped design the experiments and contributed reagents. DMW helped with data analysis and built the islet dispersion and reaggregation scheme in Figure 5. MA, ZS, JW and PM contributed human islets and reagents. MB evaluated all patients undergoing total pancreatectomy with islet auto-transplantation before and after surgery/islet transplants and obtained consent from patients to use their islets for experimentation. EPT and ML wrote the manuscript. EPT, OS, ER, and ML revised and edited the manuscript.
