# Supplementary material for: Individual islet respirometry reveals functional diversity within the islet population of mice and human donors
Source: Mol Metab. 2018 Jul 25;16:150–9. doi: 10.1016/j.molmet.2018.07.003 (PMC6157638; doi:10.1016/j.molmet.2018.07.003)
Supplement: Multimedia component 1 [file mmc1.docx]

**Appendix A. Supplementary Information**

Individual islet respirometry reveals functional diversity within the islet population of mice and human donors

Evan P. Taddeo^1^, Linsey Stiles^1,7^, Samuel Sereda^2,7^, Eleni Ritou^1^, Dane M.Wolf^1^, Muhamad Abdullah^3^, Zachary Swanson^3^, Josh Wilhelm^3^, Melena Bellin^4^, Patrick McDonald^5^, Kacey Caradonna^6^, Andrew Neilson^6^, Marc Liesa^1*^, and Orian S. Shirihai^1,2*^

^1^Department of Medicine, Division of Endocrinology Diabetes and Hypertension and Department of Molecular and Medical Pharmacology,, David Geffen School of Medicine at UCLA, 650 Charles E. Young St., CHS, Los Angeles, CA 90095, USA

^2^Department of Medicine, Endocrinology, Diabetes, Nutrition and Weight Management Section, Boston University School of Medicine, 650 Albany St., Room 840, Boston, MA 02118, USA.

^3^Department of Surgery and Schulze Diabetes Institute, University of Minnesota School of Medicine, Minneapolis, MN 55455, USA.

^4^Department of Pediatrics, Division of Pediatric Endocrinology, University of Minnesota School of Medicine, Minneapolis, MN 55455, USA.

^5^Centre for Health Research Innovation, University of Alberta, Edmonton, AB T6G 2E1, CAN.

^6^Agilent Technologies, Lexington, MA 02421, USA.

^7^ These authors contributed equally to this work.

*Shared corresponding authors at: Center for Health Sciences, 650 Charles E. Young St., CHS 27-200E, Los Angeles, CA 90095, USA. Telephone: 310-825-5160; 310-206-7139

Email: [mliesa@mednet.ucla.edu](mailto:mliesa@mednet.ucla.edu) (M. Liesa), [oshirihai@mednet.ucla.edu](mailto:oshirihai@mednet.ucla.edu) (O. Shirihai)

**Supplementary Figure 1. Validation of XF96 spheroid plate islet bioenergetics.**

(a) Representative OCR traces from large single mouse islets measured in the spheroid plate. Raw OCR (left graph) and OCR normalized to basal respiration (% Basal, right graph) are shown for three individual mouse islets. (b) Nutrient-stimulated respiration of multiple mouse islets (50-80 islets/well) measured via Seahorse XF24 or of individual mouse islets measured via Seahorse XF96 spheroid plate. Islets were acutely exposed to 20mM glucose alone or 20mM glucose + 10mM each of the amino acids leucine and glutamine. For XF24, n = 9-10 wells per nutrient from 3 independent experiments. For XF96, n = 9-12 islets per nutrient from 1 islet isolation. (c) Long-term islet nutrient stimulation. Islets were exposed to 20mM glucose or 20mM glucose + 10mM amino acids for approximately 300min. n = 4-7 individual islets per condition from one islet isolation. (d) Reproducibility of individual mouse islet measurements in the spheroid plate. After stable basal oxygen consumption measurements were achieved in the first assay, single mouse islets (red asterisk) were transferred to a new plate and placed in a different well position. Basal OCR was measured again until stable rates were achieved. Each pair of connected data points represents the last basal OCR from one islet measured in two consecutive assays. n = 11 single islets from one islet isolation. Data in (b) and (c) are means ± SEM.

**Supplementary Figure 2. Assessing mitochondrial function in different numbers of human islets with the XF96 spheroid plate.** Basal OCR (a), glucose-stimulated OCR (b), Oligomycin-resistant OCR (c), and FCCP-induced maximal respiration (d) of various sample sizes of human islets. Measurements were done with 32 islets per well down to one islet per well. n = 1-4 wells per islet number. Respiratory parameters were calculated as described in the Methods. Islets were derived from one deceased donor (University of Alberta Diabetes Institute Islet Core). Data are means ± SEM.

**Supplementary Figure 3. Images of individual human and mouse islets analyzed via XF96 spheroid plate.** (a) Operetta brightfield images (10x magnification) of single human islets of different sizes taken after Seahorse experiments. Scale bar = 200µm. The corresponding basal respiration and area are listed for each islet in the table below the images. Islets are derived from the analysis in Figure 3a. (b) Operetta brightfield images (10x magnification) of single mouse islets of different sizes taken after Seahorse experiments. Scale bar = 200µm. Basal OCR and area are listed for each islet in the accompanying table. Islets are derived from the analysis in Figure 3b. Human islets in (a) are derived from living donors with pancreatitis undergoing total pancreatectomy with islet auto-transplantation at the University of Minnesota.

**Supplementary Figure 4. Bioenergetics of large individual human and mouse islets.** (a) Glucose-stimulated respiration, Oligomycin-induced proton leak and FCCP-stimulated maximal respiration from large-sized individual human islets measured with the XF96 spheroid plate. All bioenergetics parameters were calculated after subtracting non-mitochondrial OCR. n = 18 islets from 4 donors. (b) Glucose-stimulated respiration, proton leak and maximal respiration from individual mouse islets measured with the spheroid plate. Maximal respiration of mouse islets was measured in the presence of FCCP and 10mM leucine/10mM glutamine. Bioenergetics parameters were calculated as noted in (a). n = 16-55 islets per condition, from two independent experiments. Human islets in (a) were derived from deceased donors (University of Alberta Diabetes Institute Islet Core). Data are means ± SEM.

**Supplementary Figure 5. Insulin secretion from individual mouse islets in the XF96 spheroid plate.** Insulin secretion from individual intact mouse islets. Islets were seeded in 3mM glucose Seahorse media in wells of an XF96 spheroid plate and acutely exposed to Seahorse media containing 3mM glucose or 20mM glucose (final concentration). After approximately 50-75min, media was harvested from each well for measurement of insulin concentration via Homogeneous Time Resolved Fluorescence (HTRF) assay, as described in the Supplementary Materials and Methods. n = 67 islets for 3mM glucose and 65 islets for 20mM glucose, from 4 independent experiments. Each data point represents one islet. Black lines denote means ± SEM.

**Supplementary Figure 6. Analysis of reaggregated islets with the XF96 spheroid plate.** (a) Correlation between basal respiration and size of reaggregated mouse islets consisting of 6,000, 8,000 or 10,000 islet cells. n = 6-7 individual islets for each reaggregated islet size. Correlation (R^2^) between islet size and basal OCR was assessed via linear regression, where *p < 0.05 for a non-zero slope. (b) Statistical parameters for estimating variability in glucose-stimulated respiration from intact and reaggregated (Reagg) human and mouse islets. IQR is the interquartile range (75^th^ percentile – 25^th^ percentile). Data in (a) are means ± SEM.

**Supplementary Materials and Methods**

*Individual Islet Insulin Secretion*

Individual intact mouse islets were seeded in 3mM glucose Seahorse media in wells of a PDL-coated XF96 spheroid plate, as described in 2.4. Islets were acutely exposed to Seahorse media containing 3mM glucose (basal insulin secretion) or 20mM glucose (final concentration, glucose-stimulated insulin secretion) for approximately 50-75min. Media (90µL/well) was harvested and mixed with 10µL of 5% fatty acid-free BSA (final BSA concentration of 0.5%) in 96 well plates and stored at -20 °C.

A Homogeneous Time Resolved Fluorescence (HTRF) insulin assay kit (CisBio) consisting of a FRET-based sandwich immunoassay with Europium Cryptate (Eu-K) and allophycocyanin (XL665) antibodies was used to quantify insulin levels in media. Media samples were thawed at room temperature and diluted in Krebs buffer (119mM NaCl, 20mM HEPES, 4.6mM KCl, 1mM MgSO_4_, 0.15mM Na_2_HPO_4_, 0.4mM KH_2_PO_4_, 5mM NAHCO_3_, 2mM CaCl_2_, 0.5% BSA, pH 7.4). Diluted sample (10µL) and a 1:1 Eu-K/XL antibody mix (10µL) were dispensed into white-walled 384-well plates (Greiner Bio) and incubated overnight at room temperature in the dark. A Tecan Infinite M1000 or Spark 10M plate reader was used to measure time-resolved fluorescence at emission wavelengths of 665nm (XL665) and 620nm (Eu-K). Insulin concentration of the samples was calculated from an insulin standard curve.
